# Poly(ionic liquid) Nanovesicle-Templated Carbon Nanocapsules Functionalized with Uniform Iron Nitride Nanoparticles as Catalytic Sulfur Host for Li–S Batteries

**DOI:** 10.1021/acsnano.2c01992

**Published:** 2022-07-05

**Authors:** Dongjiu Xie, Yaolin Xu, Yonglei Wang, Xuefeng Pan, Eneli Härk, Zdravko Kochovski, Alberto Eljarrat, Johannes Müller, Christoph T. Koch, Jiayin Yuan, Yan Lu

**Affiliations:** † Department for Electrochemical Energy Storage, Helmholtz-Zentrum Berlin für Materialien und Energie, Hahn-Meitner Platz 1, 14109 Berlin, Germany; ‡ Institute of Chemistry, University of Potsdam, Karl-Liebknecht-Straße 24-25, 14476 Potsdam, Germany; § Institut für Physik and IRIS Adlershof, Humboldt-Universität zu Berlin, 12489 Berlin, Germany; ∥ Department of Materials and Environmental Chemistry, Stockholm University, Stockholm 10691, Sweden

**Keywords:** poly(ionic liquid)s, nanovesicles, sulfur host, iron nitride, Li−S batteries

## Abstract

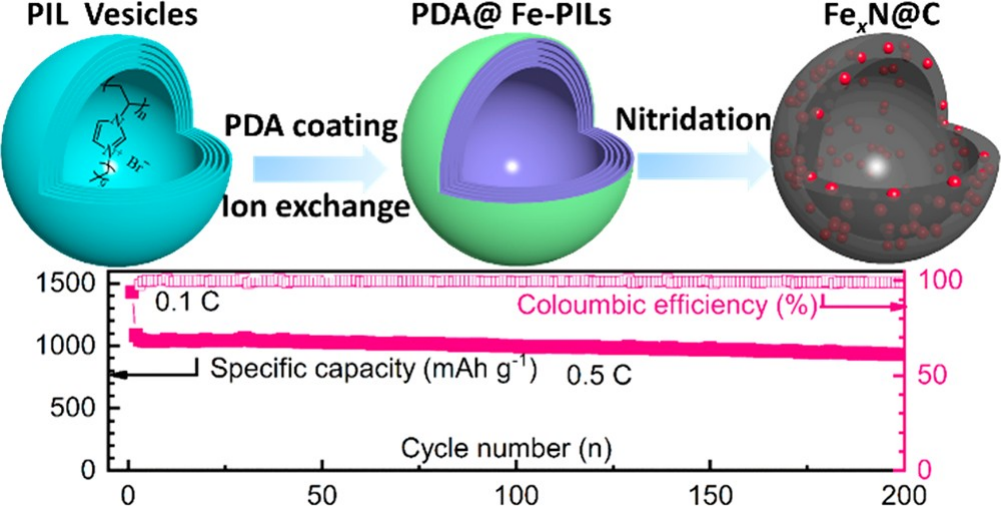

Poly(ionic liquid)s (PIL) are common precursors for heteroatom-doped carbon materials. Despite a relatively higher carbonization yield, the PIL-to-carbon conversion process faces challenges in preserving morphological and structural motifs on the nanoscale. Assisted by a thin polydopamine coating route and ion exchange, imidazolium-based PIL nanovesicles were successfully applied in morphology-maintaining carbonization to prepare carbon composite nanocapsules. Extending this strategy further to their composites, we demonstrate the synthesis of carbon composite nanocapsules functionalized with iron nitride nanoparticles of an ultrafine, uniform size of 3–5 nm (termed “Fe_*x*_N@C”). Due to its unique nanostructure, the sulfur-loaded Fe_*x*_N@C electrode was tested to efficiently mitigate the notorious shuttle effect of lithium polysulfides (LiPSs) in Li–S batteries. The cavity of the carbon nanocapsules was spotted to better the loading content of sulfur. The well-dispersed iron nitride nanoparticles effectively catalyze the conversion of LiPSs to Li_2_S, owing to their high electronic conductivity and strong binding power to LiPSs. Benefiting from this well-crafted composite nanostructure, the constructed Fe_*x*_N@C/S cathode demonstrated a fairly high discharge capacity of 1085 mAh g^–1^ at 0.5 C initially, and a remaining value of 930 mAh g^–1^ after 200 cycles. In addition, it exhibits an excellent rate capability with a high initial discharge capacity of 889.8 mAh g^–1^ at 2 C. This facile PIL-to-nanocarbon synthetic approach is applicable for the exquisite design of complex hybrid carbon nanostructures with potential use in electrochemical energy storage and conversion.

There is a persistent pursuit of cost-effective energy storage and conversion devices of high energy density for the large-scale deployment of electric vehicles and intermittent renewable energy sources. In this context, lithium–sulfur (Li–S) batteries are recognized as a potential next-generation solution to meet these requirements due to their large theoretical capacity (1675 mAh g^–1^) and natural abundance, environmental friendliness, and low cost of sulfur.^1,2^ Despite these advantages, the practical use of Li–S batteries has been hindered by the notorious shuttle effect of lithium polysulfides (Li_2_S_*n*_, 4  ≤  *n* ≤ 8), as the intermediates during cycling, which are highly soluble in the ether-based electrolyte.^3,4^ The shuttle behavior of Li_2_S_*n*_ between the cathode and anode decreases the capacity and cycling stability and raises safety concerns,^5,6^ especially when metallic lithium is used as anode.^7,8^ The multiphase conversion reactions from insulating sulfur to nonconductive Li_2_S suffer sluggish kinetics,^9^ which results in a poor rate capability and inefficient utilization of sulfur.^10,11^ In parallel, there is a large volumetric expansion of ∼80% during the sulfur-to-Li_2_S reaction on the cathode side, weakening the adhesion of the electrode components to the current collector that gives a fast capacity fading.^12^

To tackle the aforementioned issues, efforts have been devoted to improving the electrochemical performance of Li–S batteries. Significant progress has been made via impregnating sulfur into a vast variety of types of hollow as well as porous carbon-based nanomaterials with different morphologies, such as nanospheres,^13,14^ nanocapsules,^15,16^ nanotubes,^17,18^ and nanocages.^19−21^ For example, Lyu et al. prepared three-dimensional hierarchical carbon nanocages as sulfur host material with MgO as the template, which could alleviate the polysulfide dissolution issue and enhance the electron conduction and Li-ion diffusion.^19^ However, it remains challenging for nanostructured carbon-based materials to effectively catalyze the multiple-step conversion reactions from LiPSs to Li_2_S. Therefore, the confinement of LiPSs on-site within electrocatalytic host materials is considered the most efficient approach, since the full utilization of sulfur can be realized only when the confinement and catalytic conversion of LiPSs are integrated simultaneously.^11,22^ Considering the insulating character of sulfur and polar LiPSs, nanostructured carbon/metal composites are needed and regarded as an all-in-one sulfur host to offer physical confinement, conductive matrix, and chemical adsorption.^23,24^ To strengthen the interaction between the host materials and LiPSs guest, a wide range of metal compounds have been examined extensively as the LiPSs mediator, including metal oxides,^25−27^ nitrides,^28,29^ sulfides,^22^ carbides,^30^ phosphides,^31,32^ and selenides.^33,34^ Among them, transition-metal nitrides (TMNs) are of particular interest as electrocatalysts for the conversion of polysulfides because of their merits of superior electrical conductivity, sufficient chemical stability, and polar metal–nitrogen (M–N) bonds.^35,36^ Some TMNs have been previously investigated as sulfur host materials in Li–S batteries, such as TiN,^37,38^ VN,^29,39,40^ InN,^41^ Co_4_N,^42−44^ MoN,^45,46^ and WN.^47^ For instance, Cui et al. reported a mesoporous TiN as sulfur host materials, in which the TiN-S composite cathode delivered a capacity of over 644 mAh g^–1^ after 500 cycles at 0.5 C.^38^ Sun et al. developed a mesoporous VN nanorod and graphene composite, exhibiting lower polarization and faster redox reaction kinetics than that of the reduced graphene oxide cathode.^29^ Meanwhile, it has been noticed that those metal-based nitride nanoparticles often suffered from high cost, low surface area, low utilization of catalytic particles, and lack of voids or space to accommodate a sufficiently large amount of sulfur.

Consequently, earth-abundant and environmentally friendly iron-based nitrides have moved into the frontline of the electrochemical field due to their high electronic conductivity and catalytic activity. Recently, yolk-shelled Fe_2_N-carbon nanoboxes were designed by Sun et al. as sulfur host materials for Li–S batteries, and the polar iron nitride (Fe_2_N) core could provide strong chemical bonding and effective catalytic activity for polysulfides.^48^ Later on, Zhang et al. reported that phosphorus doping could boost the catalytic activity of the iron nitride (Fe_4_N) nanoparticles.^49^ However, the particle size of these iron nitrides was big, that is, in the range of 30–100 nm, thus suppressing the better utilization efficiency of the catalyst. Ideally, well-dispersed sub-10 nm metal-based nanoparticles could immediately expose at least 10 times more active sites for boosting the catalytic, electronic, and kinetic performance.^50,51^ However, when synthesized from nanosized precursor particles, they typically tend to agglomerate and grow adversely into much larger ones during calcination.^50^ Usually, time-consuming and complicated preparative routes are required, for example, the template-assisted selective etching fabrication or postloading methods.

To address this challenge, polymer–metal ion complexes as the precursor to ultrafine metal nanoparticles via one-step calcination have been actively attempted, since the parallel polymer-to-carbon conversion could in situ produce a carbonaceous matrix to impede or slow down the growth kinetics of these nanoparticles.^50,52^ In this regard, imidazolium-based poly(ionic liquid)s (PILs) with N-rich ionic liquids as repeating units have been of great interest, not only because they can form N-doped porous carbon fibers or membranes with controllable N content and conductivity but also the possible coordination with the metallic species via the nitrogen atoms during pyrolysis.^53^ Equally important, the introduction of metal ions into PILs can be fairly easy via the ion-complexation or ion-exchange method. Unsurprisingly, imidazolium-based PIL could produce nanostructured carbon materials embedded with metal-containing species.^54−56^ Chen et al. reported carbon nanosheets with small cobalt nanoparticles by using PIL-cobaltinitrite complex/graphene oxide as precursors, which were then used to modify the separator membrane for the Li–S battery.^55^ Nevertheless, due to a high density of ionic liquid species, PIL-derived carbon materials face challenges in maintaining PIL’s morphology on the nanoscale. The state-of-the-art methods rely much on the time-consuming hard-template (silica) coating or silica nanocasting process.^57−59^

In this study, we developed a facile synthetic route toward structurally complex, PIL nanovesicle-templated carbon composite nanocapsules, which contain ultrafine iron nitride nanoparticles of 3–5 nm in size embedded within the carbon nanocapsules. Assisted by a protective polydopamine (PDA) coating in combination with an ion-exchange process to introduce the iron species, the hollow spherical morphology of PIL nanovesicles has, despite an inevitable dimensional shrinkage to some extent due to a large weight loss, been preserved along with pyrolysis and successfully transferred into the functional composite product. The designed nanocomposite has served as an efficient sulfur host material for Li–S batteries with drastically improved electrochemical performance. The catalytic and conductive iron nitride nanoparticles possessed abundant active sites to assist LiPS conversion and Li_2_S nucleation during cycling. Besides, our synthetic route could be applied to fabricate other metal compounds (nitrides, sulfides, and phosphides) with similar nanostructures for a broader range of electrochemical applications, that is, in fuel cells and supercapacitors.

## Results and Discussion

**Figure 1:**
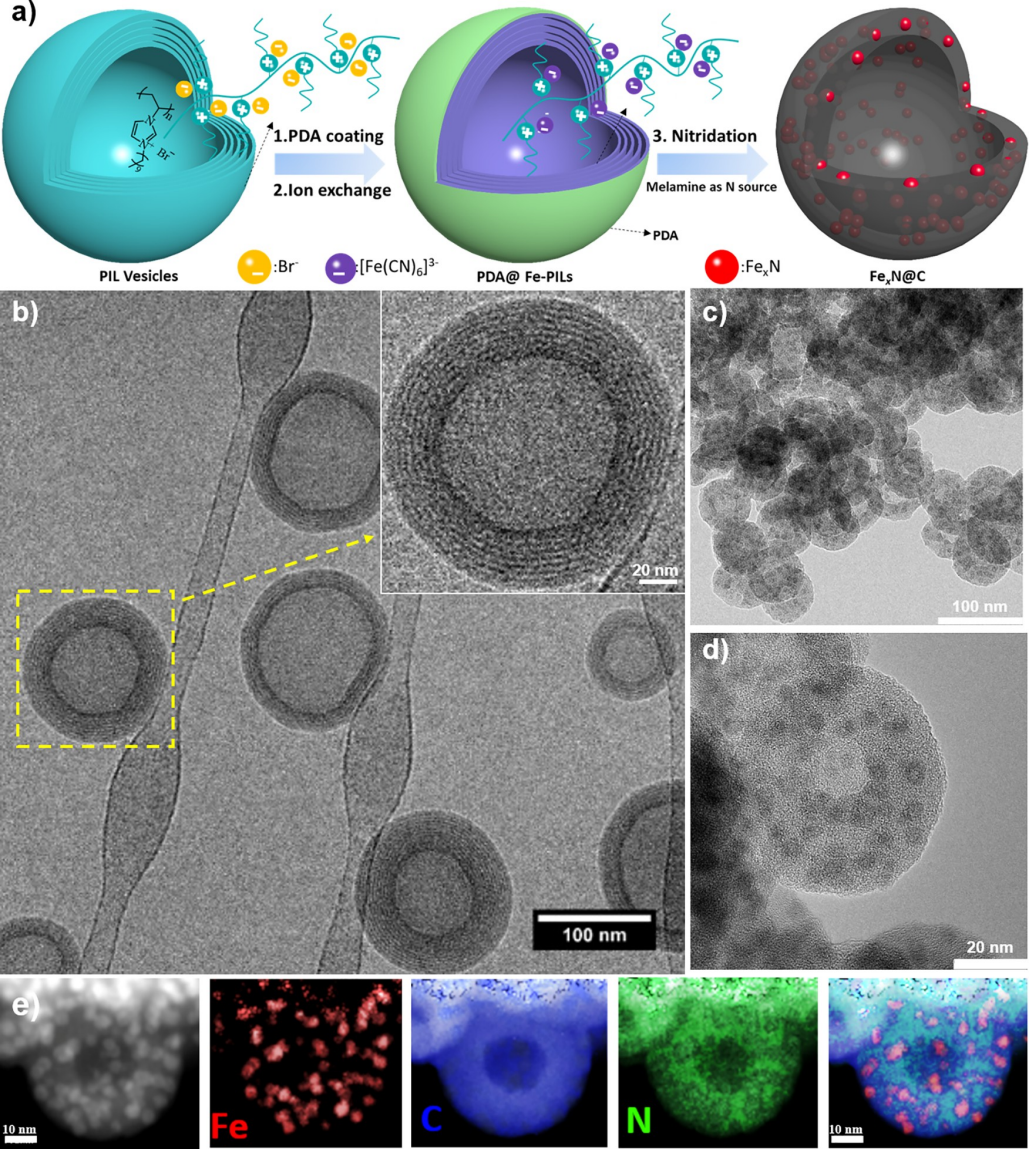
(a) Schematic representation of the synthesis route to Fe_*x*_N@C nanocapsules. (b) Cryo-TEM images of the PIL nanovesicles; (c and d) TEM images of the Fe_*x*_N@C nanocapsules. (e) STEM image and corresponding EELS mapping images of the Fe_*x*_N@C nanocapsules.

The scheme in Figure 1a describes the synthetic route to the target composite, that is, iron nitride nanoparticle-functionalized carbon nanocapsules. First, PIL nanovesicles were synthesized by one-step radical homopolymerization of the monomer 3-*n*-decyl-1 vinylimidazolium bromide (ILM-10) in water at 75 °C using VA-86 as initiator under nitrogen atmosphere. After purification by dialysis, a stable PIL colloidal dispersion was obtained with a solid concentration of 10 g L^–1^. The PIL nanoparticles exhibit a characteristic vesicular shape, as sketched in Figure 1b, with an outer diameter of 100 ± 10 nm. Interestingly, the PIL nanovesicle consists of several repeated lamellas in its wall. The alternating light and dark nanodomains stem from the hydrophobic alkyl chains and the charged backbones, respectively, due to a higher electron density of the bromide anions than that of the alkyl chains.^60^ The distribution of the bromide anions (the dark lamellae) across the entire wall is a beneficial feature, as they will be anion-exchanged with ferricyanide that will be also evenly dispersed all over the wall. Besides, the SEM image of the PIL nanovesicles is further demonstrated in Figure S1. It is found that after the drying process, the morphology of some PIL nanovesicles is partially deviated from their original spherical shape, and occasionally a bowl-like structure can be observed. According to the previous report, the deformation of certain vesicles is caused expectedly by the loss of the structural water component as well as the softness of the PIL outer layer to withstand the capillary force exerted on the vesicles.^61,62^ To avoid ionic cross-linking of the PIL nanovesicles possibly by ferricyanide during anion exchange, a thin protective layer of PDA was coated by in situ polymerization onto the surface of the PIL nanovesicles. The existence of the PDA layer can help preserve the nanomorpholoy of PIL during calcination because the fluidic fragmentation intermediates of PIL during carbonization may cause the amalgamation of individual PIL nanovesicles together to forfeit their nanoscopic morphology.^63^ As shown in Figure S2, after deposition of a PDA layer in a Tris HCl buffer (pH = 8.5) aqueous solution, PDA@PIL dispersion turned dark, and the multilamellar pattern in the wall of the PIL nanovesicle remains (Figure S3a). Due to the dynamic feature of ionic bonds, ion exchange has been widely applied to introduce alien metal atoms into PIL.^55,56^ Here, the anionic ferricyanide ions were chosen to exchange with bromide ions in the polyimidazolium-based vesicles. As depicted in Figure S4, the element mapping images of the anion-exchanged product, PDA@Fe-PIL nanovesicles, visualize the homogeneous distribution of iron atoms with a low Br atom content, indicating the successful introduction of iron-containing anions into the PIL nanovesicles. Besides, after anion exchange, there is no noticeable change in the PIL vesicle structure as depicted in the cryo-transmission electron microscopy (TEM) (Figure S3b) and scanning electron microscopy (SEM) image (Figure S5a).

**Figure 2:**
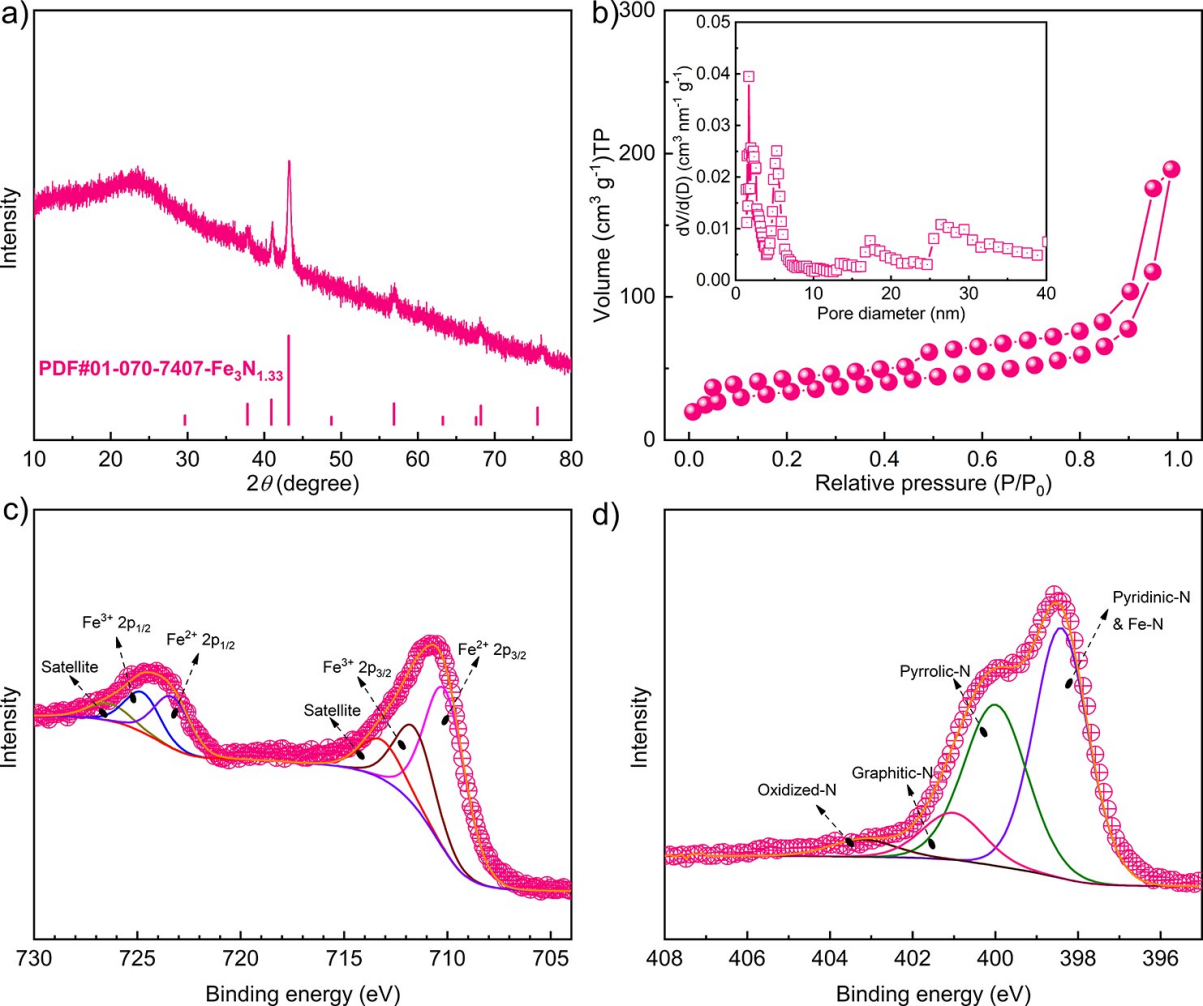
(a) XRD pattern, (b) nitrogen adsorption–desorption isotherms with the inset corresponding to the pore size distribution plot. XPS spectra of (c) Fe 2p and (d) N 1s of the sample Fe_*x*_N@C.

After the nitridation, the morphology of the Fe_*x*_N@C sample was visualized by SEM and TEM. The SEM image in Figure S5b supports that the Fe_*x*_N@C particles inherit a similar morphology to the PIL vesicle template. The TEM images of the Fe_*x*_N@C sample in Figure 1c,d show the as-prepared hybrid carbon product, here the visible nanocapsules of ∼50 nm in diameter and 15–25 nm in thickness and the fine iron nitride nanoparticles of 3–5 nm in size are found well-dispersed all over the hollow carbon shell. Furthermore, a scanning transmission electron microscopy (STEM) image of the Fe_*x*_N@C sample with the corresponding electron energy-loss spectroscopy (EELS) elemental mapping of Fe, C, and N is demonstrated in Figure 1e. It is found that the N-content near the carbon nanocapsule inner layer is higher than that of the outer layer. This observation could be ascribed to the imidazole-based PILs as the carbon source, which could preserve a higher N content than that of PDA. The crystalline structure of Fe_*x*_N@C calcinated at 500 °C is further characterized by X-ray diffraction (XRD) and HRTEM. The XRD pattern in Figure 2a can be well-indexed to the hexagonal phase of iron nitride (Fe_3_N_1.33_, PDF01-070-7407). The lattice fringes of the iron nitride particles are clearly observed in the HRTEM images (Figure S6). The interplanar distance is measured to be 0.31 nm, corresponding to the (101) facet of Fe_3_N_1.33_.^64^ The effect of the calcination temperature on the formation of iron nitride particles has been studied, and the results are presented in Figure S7. It is found that the formation of the iron nitride phase is temperature-specific, that is, it can only be obtained at 500–600 °C. Beyond 700 °C, a metallic iron phase emerges. Moreover, Figure S8 reveals that the iron nitride obtained at 600 °C exhibits a larger particle size of 20–70 nm than that at 500 °C. From here on, only the iron nitride obtained at 500 °C was used in the rest work.

The effect of PDA coating, ferricyanide ions, and melamine on the morphology of the PILs nanovesicles-derived carbon calcinated at 500 °C was investigated systematically (Figure S9). Obviously, without a PDA coating, only irregularly shaped bulk carbon embedded with metal-containing nanoparticles is obtained, as shown in Figure S9a. Similarly carbon bulk (Figure S9b) is collected when calcinating the iron-free PDA@PIL nanovesicles under the same carbonization. The irregular morphology of carbon is due to the convergence of the PIL particles in a softened state at elevated temperature and could not hold their vesicular shape. Melamine as a nitrogen source was also found important to maintain the rather small size of the iron nitride nanoparticles. Without melamine, the nanocapsule shape of carbon is still nicely obtained, but in them, only a few large iron-based particles in size of 7–20 nm are embedded (Figure S9c). Similar to a previous report, the good dispersity of iron nitride nanoparticles stems from the N species in the melamine and imidazole-ring that act as anchoring site points to the metallic species.^52^ Furthermore, the iron content inside the Fe_*x*_N@C nanocapsules is 22.7 wt %, calculated from the Fe_2_O_3_ residual based on the thermogravimetric analysis (TGA) result in Figure S10. The metal-free carbon nanocapsules (Figure S9d) have been obtained by etching the iron nitride particles out of the Fe_*x*_N@C sample via acid treatment. Based on the TEM analysis and TGA result in Figures S9 and S10, iron nitride particles are removed after the repeated acid etching process.

To measure the specific surface area and pore size distribution of Fe_*x*_N@C, the N_2_ adsorption–desorption measurement at 77 K was conducted. A hysteresis loop can be observed in Figure 2b, which resembles a typical type-III isotherm. The pore size distribution diagram in the inset of Figure 2b shows that Fe_*x*_N@C nanocapsules possess mesopores of a multimodal pore size distribution that peaks at 5, 17, and 22 nm. The 5 nm mesopores may be related to the pore inside the carbon shell, while the larger mesopores of 17 and 22 nm may stem from the hollow voids inside the carbon nanocapsules. The existence of mesopores in the Fe_*x*_N@C sample is favorable for the sulfur loading, and their surface can also serve as the physical adsorption reservoir for LiPSs, mitigating the shuttling effect. Based on the Brunauer–Emmett–Teller (BET) equation, the specific surface area of the Fe_*x*_N@C sample is 120 m^2^ g^–1^, while 233 m^2^ g^–1^ for the N-Carbon sample due to the removal of iron nitride particles that has a higher density than carbon, as shown in Figure S11. The X-ray photoelectron spectroscopy (XPS) was applied to extract structure information on the surface composition and valence state in the Fe_*x*_N@C nanocapsules. The high-resolution Fe 2p and N 1s spectra of the Fe_*x*_N@C sample are displayed in Figure 2c,d, respectively. For the Fe 2p spectrum, two peaks at 723.4 and 710.3 eV are found and assigned to Fe 2p_1/2_ and Fe 2p_3/2_ of Fe^2+^ states, respectively; the Fe 2p_1/2_ and Fe 2p_3/2_ of Fe^3+^ are found located at 724.8 and 711.8 eV, respectively.^65^ In addition, the peaks at 713.4 and 726.5 eV are satellite peaks. The coexistence of Fe^3+^/Fe^2+^ is due to the surface oxidation of the iron nitride nanoparticles when they are exposed to air. Nitrogen doping into the carbon material can provide multiple favorable effects here, that is, to enhance the electrical conductivity and offer more active sites for the chemical confinement of LiPSs.^66^ The N 1s spectrum in Figure 2d can be deconvoluted into four peaks at 398.1, 400.2, 401.1, and 403.2 eV, corresponding to the pyridinic, pyrrolic, graphitic, and oxidized N species, respectively.^67^ It is worth noting that the peak of the pyridinic N includes the contribution of the Fe–N since the binding energy of N–Fe is close to that of the pyridinic N.^68,69^

**Figure 3:**
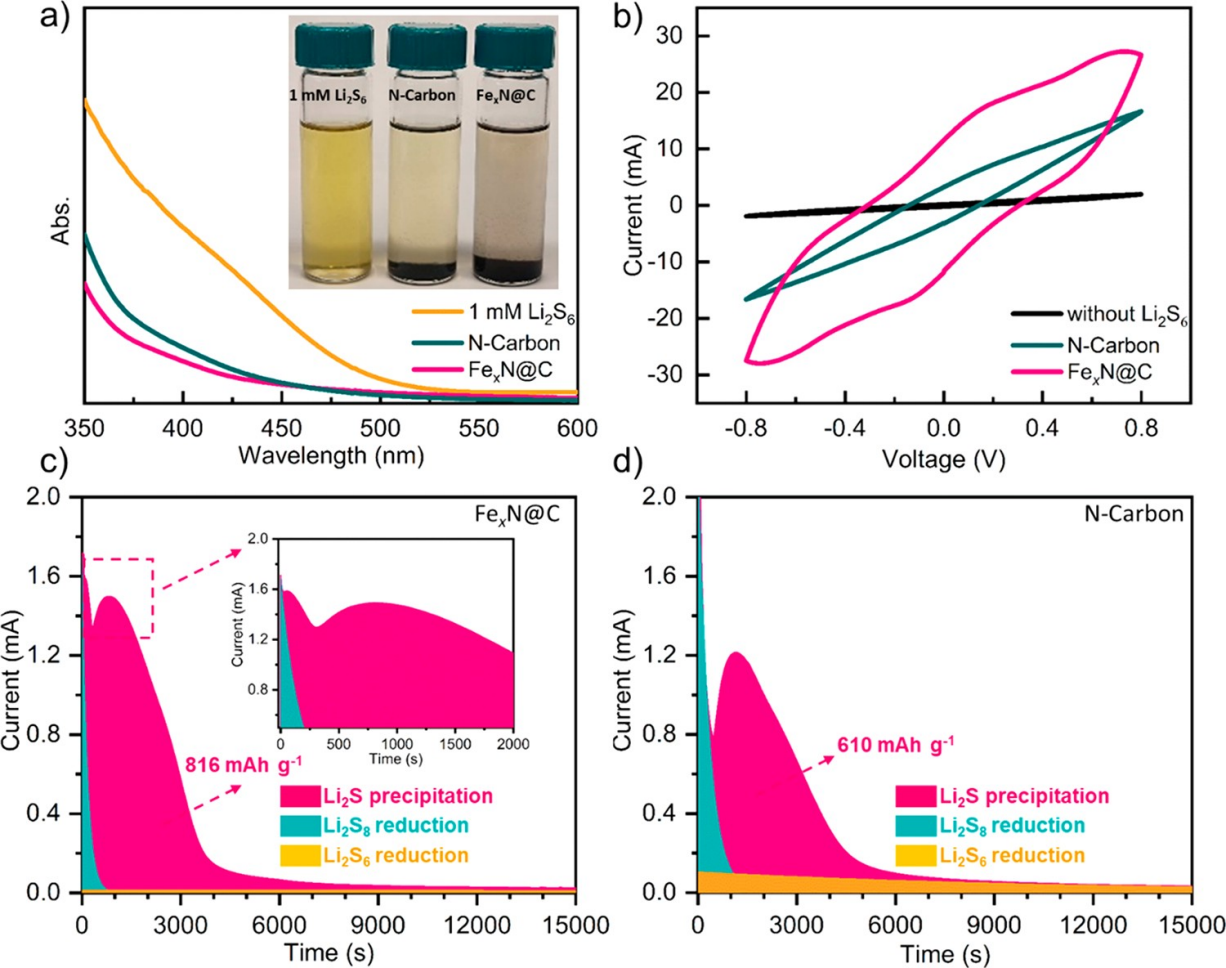
(a) Static adsorption for the Li_2_S_6_ solution by N-Carbon and Fe_*x*_N@C with the UV–vis spectra of the corresponding supernatants, respectively. (b) CV curves at a scan rate of 10 mV s^–1^ from −0.8 to 0.8 V of the symmetric batteries with different electrodes as noted in figure, with and without the presence of Li_2_S_6_. The Li_2_S precipitation test on different electrodes: (c) Fe_*x*_N@C and (d) N-Carbon.

To analyze the adsorption capability of Fe_*x*_N@C to the LiPSs, the adsorption tests were conducted. The inset of Figure 3a shows the result of the Li_2_S_6_ adsorption tests of Fe_*x*_N@C and N-Carbon particles. Six mL of Li_2_S_6_ solution (1 mM) in a mixture solvent DME/DOL (v/v = 1/1) was added into vials with different host materials (N-Carbon and Fe_*x*_N@C). To study the affinity of each material to LiPSs, the same surface area of 2 m^2^ based on the obtained specific surface area has been applied. After aging for 6 h in a glovebox, the color of the supernatant of the Fe_*x*_N@C sample turned from yellow to colorless, indicative of their strong adsorption capability to extract LiPSs from solution to solid. By contrast, the color of the supernatant with metal-free carbon nanocapsules was light yellow, suggesting a weaker affinity to LiPSs. The UV–vis spectra of the two supernatant solutions in Figure 3a were recorded, and their comparison confirms that the Li_2_S_6_ concentration in the Fe_*x*_N@C sample solution is reduced notably. To analyze the adsorption capability of Fe_*x*_N@C to the LiPSs, we performed high-resolution XPS analysis of the Fe_*x*_N@C particles before and after adsorption of Li_2_S_6_, and the results are shown in Figure S12. An obvious blue shift can be observed in the Fe 2p spectra after adsorption of Li_2_S_6_, which is highlighted with the dotted lines to show the difference between the two XPS spectra. In addition, a weak shoulder peak at 708 eV in the Fe 2p_3/2_ region of Fe_*x*_N@C–Li_2_S_6_ can be distinguished and allocated to the Fe–S binding.^70^ The blue shift in the Fe 2p spectrum of Fe_*x*_N@C–Li_2_S_6_ suggests that the iron nitride particles indeed provide a strong chemical interaction toward LiPSs.^71^ The formation of Fe–S binding in the Fe_*x*_N@C–Li_2_S_6_ indicates the electrons of sulfur atoms in the polysulfide molecules are transferred to the iron atoms, which could reduce the energy barrier for the conversion of LiPSs to Li_2_S.^72,73^

The catalytic effects of Fe_*x*_N@C on the conversion reaction of LiPSs to Li_2_S were investigated in a symmetrical test cell by cyclic voltammetry (CV) method, as shown in Figure 3b. Since the Li_2_S_6_-free symmetrical cell only exhibits a minor contribution from the capacitive current, the polarization profiles of cells with the other two electrodes are assigned to the redox current of Li_2_S_6_. It can be observed that the current density significantly increases when Fe_*x*_N@C and N-Carbon nanocapsules were used as the electrodes, demonstrating that both iron nitride and hollow carbon nanocapsules can significantly facilitate the electrochemical reactions of LiPSs. The larger area of the CV curves in the Fe_*x*_N@C electrode than that of the carbon nanocapsules suggests considerable improvement in the redox kinetics of LiPSs conversion reactions in a liquid phase (Li_2_S_8_ ↔ Li_2_S_6_ ↔ Li_2_S_4_) by introducing iron nitride nanoparticles into carbon nanocapsules.^9^ To further explore the catalytic properties of the Fe_*x*_N@C nanoparticles, Li_2_S nucleation was investigated with the potentiostat discharging method.^74^ As observed in Figure 3, the presence of Fe_*x*_N@C notably results in faster nucleation and growth and a higher discharging peak current during potentiostatic discharge at 2.05 V vs Li/Li^+^. Based on Faraday’s law, the discharge capacity of the Fe_*x*_N@C electrode was 816 mAh g^–1^, which is higher than that of the N-Carbon electrode (610.1 mAh g^–1^). Apart from this observation, the current peak position during the Li_2_S precipitation process at Fe_*x*_N@C is located at 52 s, which is much earlier than that for the N-Carbon electrode. This result suggests that iron nitride nanoparticles can boost the kinetics of the Li_2_S precipitation process during cycling.

**Figure 4:**
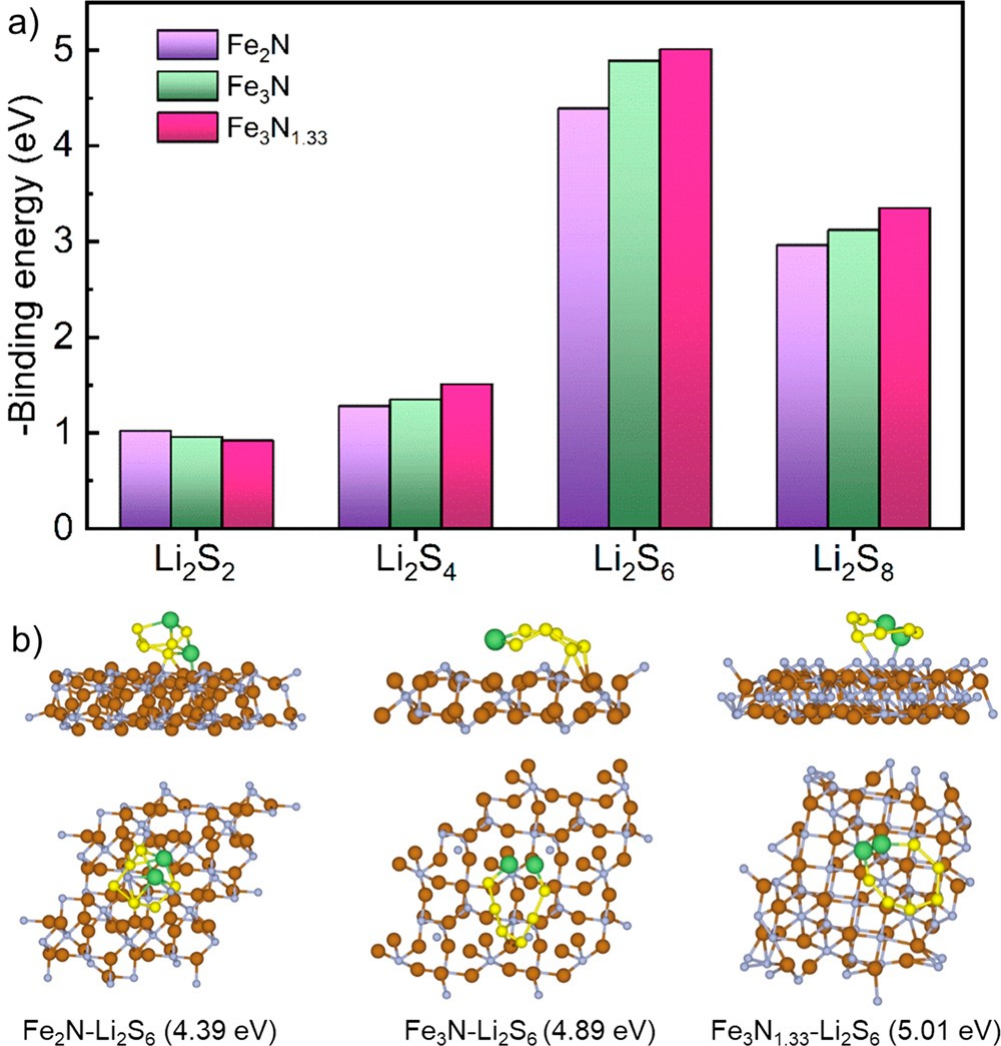
DFT calculations: (a) the binding energy of different LiPSs molecules (Li_2_S_2_, Li_2_S_4_, Li_2_S_6_, and Li_2_S_8_) on the surfaces of different iron nitrides (Fe_2_N, Fe_3_N, and Fe_3_N_1.33_). (b) The optimized binding configurations of the Li_2_S_6_ molecule on the surfaces of different iron nitrides, as noted in the figure.

Furthermore, first-principle simulations based on density functional theory (DFT) were applied to theoretically study the binding energy, structures, and motifs of LiPSs (Li_2_S_2_, Li_2_S_4_, Li_2_S_6_, and Li_2_S_8_) molecules on the surfaces of three common iron nitrides (Fe_2_N, Fe_3_N, and Fe_3_N_1.33_). The binding energy of LiPSs (Li_2_S_*n*_ with *n* = 2, 4, 6, and 8) on varied iron nitride surfaces (Fe_2_N, Fe_3_N, and Fe_3_N_1.33_) was calculated via the formula *E* = *E*_Li_2_S_*n*_–Fe*_x_*N complexes_ – *E*_Li_2_S_*n*__ – *E*_Fe*_x_*N_, in which *E*_Li_2_S_*n*__, *E*_Fe*_x_*N_, and *E*_Li_2_S_*n*_–Fe*_x_*N_ complexes are the total energies of Li_2_S_*n*_ molecules, iron nitride surfaces, and the adsorption of Li_2_S_*n*_ on iron nitride surfaces with optimized adsorption structure, respectively. A more negative value indicates a much stronger adsorption capability of LiPSs species on the given iron nitride surfaces. As demonstrated in Figure 4a and Figure S13, the calculated binding energies for Li_2_S_*x*_ species with a given iron nitride can be described by Li_2_S_6_ > Li_2_S_8_ > Li_2_S_4_ > L_2_S_2_, and the iron nitride Fe_3_N_1.33_ exhibits a better adsorption capability to long-chain LiPSs than the other two ones. For instance, the calculated surface adsorption energies for a Li_2_S_6_ molecule on the iron nitrides are 4.39, 4.89, and 5.01 eV for Fe_2_N, Fe_3_N, and Fe_3_N_1.33_, respectively. It is worth noting that the obtained binding energies of different LiPSs on Fe_2_N are well consistent with those determined from previous DFT calculations,^48^ indicating the good reliability of the applied calculated methods. The higher binding energy indicates a better chemical trapping capability of soluble Li_2_S_6_ and Li_2_S_8_ species on the Fe_3_N_1.33_ electrode, which could substantially mitigate the shuttle effect of LiPSs. A further inspection of the optimized binding structures of the Li_2_S_6_ molecule on the Fe_3_N_1.33_ demonstrates that the Li_2_S_6_ molecule is more likely to coordinate with the nitrogen atoms on the iron nitride through the interaction between lithium and nitrogen. In contrast, the sulfur atoms of Li_2_S_6_ have strong interactions with the central Fe atoms surrounded by N atoms. Besides, Li_2_S_6_ molecules on the iron nitride surfaces tend to be curved or bent rather than stretched, which could be favorable for breaking long polysulfides into shorter ones during cycling.

**Figure 5:**
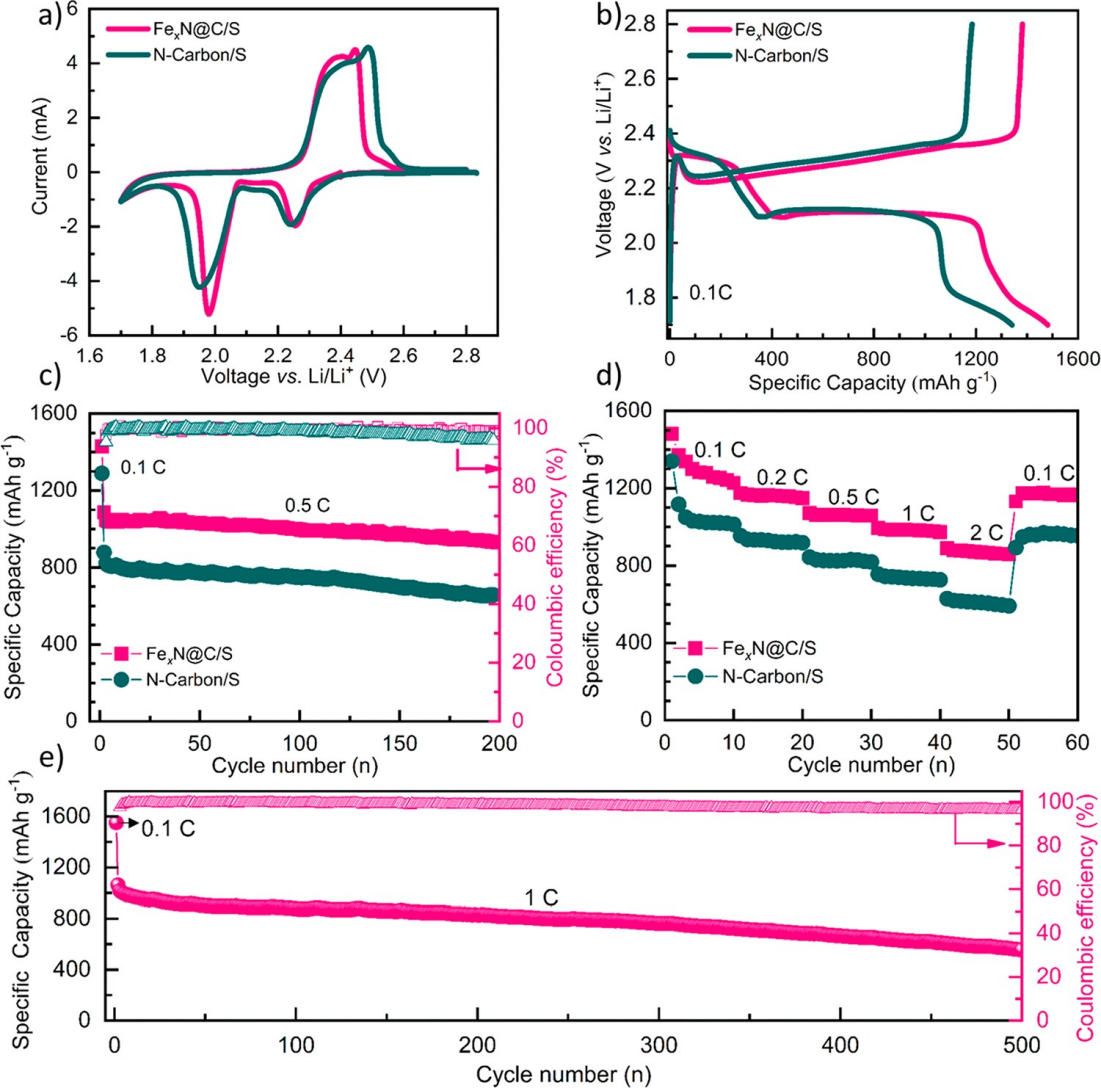
(a) CV curves scanned at 0.1 mV s^–1^, (b) discharge–charge curves at 0.1 C (1 C = 1675 mA g^–1^), (c) cycling stability at 0.5 C, and (d) rate capability of the Li–S batteries with Fe_*x*_N@C/S and N-Carbon/S as the cathode. (e) Long-term cycling performance at 1 C of the Li–S batteries with Fe_*x*_N@C/S as the cathode. All measurements have been performed in the range of 1.7–2.8 V vs Li/Li^+^.

To demonstrate the structural merits of the hollow Fe_*x*_N@C, a sulfur-loaded composite of Fe_*x*_N@C/S was prepared by the melting diffusion method and used as cathode materials for Li–S batteries. The specific sulfur contents inside the composites are 72.1 wt % for Fe_*x*_N@C/S and 72.6 wt % for N-Carbon/S, respectively, which are determined by TGA (Figure S14). The CV measurements were conducted in a cell voltage range of 1.7–2.8 V vs Li/Li^+^ at a scanning rate of 0.1 mV s^–1^. Two representative sharp peaks are observed in the first cathodic scan as shown in Figure 5a. The first peak around 2.25 V corresponds to the reduction of sulfur to long-chain LiPSs (Li_2_S_*n*_), and the other is ascribed to the reduction of LiPSs to short-chain lithium sulfides (Li_2_S_2_/Li_2_S). During the successive anodic scanning, the two oxidation peaks (2.39 and 2.44 V) can be assigned to the reversible conversion of Li_2_S to LiPSs and finally to sulfur. To note, the peak position of Fe_*x*_N@C in the cathodic scan is shifted to a higher electrode potential, while in the anodic scan, it moves to a lower electrode potential, indicating the lower polarization of the battery with Fe_*x*_N@C as the host materials compared with the carbon nanocapsules. This could be ascribed to the high conductivity and the catalytic effect of the ultrafine iron nitride particles inside the carbon nanocapsules. The initial charging–discharging curves of these two electrodes at 0.1C are given in Figure 5b. The initial specific discharge capacity of the Fe_*x*_N@C/S electrode is 1481.5 mAh g^–1^, which is much higher than that of the N-Carbon/S (1345 mAh g^–1^). Two characteristic plateaus in the discharge curve are observed at around 2.3 and 2.1 V for both studied electrodes, corresponding to the conversion reactions from sulfur to LiPSs and their further reduction to lithium sulfide, respectively. The cycling performance of Li–S batteries with different sulfur host materials was further measured at 0.5 C (Figure 5c). The initial discharge capacity of the N-Carbon/S electrode was 878.9 mAh g^–1^ at 0.5 C. After 100 cycles, it still capable of delivering a capacity of 748.8 mAh g^–1^, which is much higher than that of the previous carbon nanocapsule/sulfur composites.^15^ This could be ascribed to nitrogen-rich carbon derived from PDA and PILs, which can offer rich sites to chemically anchor LiPSs. Combined with the synergistic chemical adsorption and catalytic effect of iron nitride particles and the physical confinement of the carbon nanocapsules to suppress the shuttle effect, the Fe_*x*_N@C/S electrode shows excellent cycling stability and delivers a high initial discharge capacity of 1085 mAh g^–1^ at 0.5 C with a remaining value of 930 mAh g^–1^ after 200 cycles, which is much higher than its counterparts. To confirm the structural robustness of the nanocapsule particles, the Fe_*x*_N@C/S electrode after cycling was washed with CS_2_ and ethanol and imaged with TEM. As shown in Figure S15, the Fe_*x*_N@C particles still maintained nanocapsular morphology without breaking down, suggesting good structural robustness.

The rate capabilities of Fe_*x*_N@C/S and N-Carbon/S electrodes were measured at different current densities and displayed in Figure 5d. After 10 cycles at each current, the retained discharge capacities of Fe_*x*_N@C/S electrode are 1226.9, 1148.7, 1057.6, 973.8, and 858.1 mAh g^–1^at 0.1, 0.2, 0.5, 1, and 2 C, respectively. Under the same conditions, the N-Carbon/S electrode delivers a remaining capacity of 1012.5, 918.0, 818.9, 725.4, and 591.4 mAh g^–1^ at 0.1, 0.2, 0.5, 1, and 2 C, respectively. Since the iron nitride particles inside carbon nanocapsules can facilitate the conversion reaction from LiPSs to Li_2_S, it is expected that the Fe_*x*_N@C/S electrode exhibits a better rate capability than that of the N-Carbon/S electrode. Meanwhile, the EIS results in Figure S16 prove that the coin cell with Fe_*x*_N@C/S electrode exhibits a lower charge-transfer resistance than that with N-Carbon/S, which could be contributed to its better rate capability. From the charge/discharge voltage profiles of the Fe_*x*_N@C/S electrode at different current densities in Figure S17, the charge platforms shifted positively, and the discharge platforms shifted negatively with the increasing rate, which is attributed to the polarization. Moreover, the two typical discharge plateaus are present even when the current density increases to 2 C, suggesting the fast redox kinetics of the Fe_*x*_N@C electrode due to the high conductivity and catalytic effect of iron nitride particles. Finally, the long-term cycling stability of the Li–S battery with the Fe_*x*_N@C/S electrode has been tested and demonstrated in Figure 5e. After the activation at 0.1 C for one cycle, the Fe_*x*_N@C/S electrode shows an initial discharge capacity of 1061 mAh g^–1^ at 1 C, which is higher than that of the recently reported metal nitrides summarized in Table S1. Although direct comparison in electrochemical performances among different systems is not reasonable due to the different fabrication parameters, the comparable results in this work suggest our unique composite nanostructure with pregnated fine iron nitride particles could improve the sulfur utilization efficiency even at a high C-rate. After 500 cycles, it continues to deliver a discharge capacity of 556.5 mAh g^–1^ with an average fading rate of 0.095% per cycle, indicating the potential of Fe_*x*_N@C nanocapsules to promote the applications of Li–S batteries.

## Conclusions

In summary, structurally exquisite carbon nanocapsules were successfully templated from morphologically similar poly(ionic liquid) nanovesicles assisted by the PDA coating and ion exchange, a method to prepare their carbon/iron nitride composite nanocapsules. Because of the unique hollow nanostructure, the obtained carbon nanocapsules embedded with ultrafine iron nitride nanoparticles worked as an efficient sulfur host material for the Li–S battery. The large voids in the nanocapsules accommodate a large amount of sulfur loading (70 wt %) and mitigate the volumetric expansion during the cycling process. The iron nitride nanoparticles were found to significantly facilitate the LiPSs to Li_2_S conversion during the discharging process due to their high conductivity and catalytic activity. Based on the DFT calculation and adsorption test, Fe_*x*_N@C nanocapsules exhibited a strong ability to confine LiPSs due to a high chemical binding energy of iron nitride nanoparticles and the physical confinement of carbon nanocapsules. Benefiting from those advantages, the Fe_*x*_N@C/S electrode delivered a high initial discharge capacity of 1085 mAh g^–1^ at 0.5 C with a remaining value of 930 mAh g^–1^ after 200 cycles; it also exhibited a high rate capability with the initial discharge capacity of 889.8 mAh g^–1^ at 2 C. We believe that the synthetic route developed in this work can serve to obtain structurally well-defined porous carbon nanomaterials functionalized with metal-based compounds, and they are expected to meet electrochemical applications beyond batteries.

## Methods

### Materials

Sublimed sulfur powder, lithium nitrate (LiNO_3_), dopamine hydrochloride, potassium ferricyanide (III), anhydrous ethanol, bis(trifluoromethane)sulfonimide lithium salt (LiTFSI), polyvinylidene fluoride (PVDF), *N*-methyl-2-pyrrolidone (NMP), 1-vinylimidazole (≥99%), 1-bromodecane (98%), 1,2-dimethoxyethane (DME), carbon disulfide, tris(hydroxymethyl) aminomethane (Tris), and 1,3-dioxolane (DOL) were purchased from Sigma-Aldrich. Hydrochloric acid solution (37%) and melamine were purchased from Alfa-Aesar. Water-soluble nonionic azo initiator 2,2′-azobis[2-methyl-*N*-(2-hydroxyethyl) propionamide] (VA086) was obtained from Wako Chemicals. All chemicals were used without any further purification.

### Synthesis of Ionic Liquid Monomer and Hollow PIL Nanocapsules

According to the previous report,^58^ the monomer 3-*n*-decyl-1-vinylimidazolium bromide (ILM-10) was synthesized by dissolving 0.1 mol of 1-vinylimidazole and 0.1 mol of decyl bromide into 30 mL of methanol. Then, the mixture was stirred at 60 °C for 15 h. After cooling down, the reaction mixture was added dropwise into 1 L of diethyl ether. The white precipitate was filtered off and dried under vacuum at room temperature. The polymerization of ILM-10 monomer to form hollow PIL nanovesicles was based on our previous report with minor changes.^75^ Specifically, 1 g of the monomer ILM-10 and 150 mg of water-soluble initiator VA086 were dissolved in 100 mL of water inside a 250 mL Schlenk flask. The air inside the Schlenk flask was replaced by argon by three freeze–pump–thaw cycles. Afterward, it was put into an oil bath and stirred at 75 °C for 24 h. After being cooled down to room temperature, a stable translucent dispersion was obtained. The stable dispersion was then purified via dialysis against deionized water (>10-fold volume) for 3 days, replacing the water every 12 h.

### Synthesis of Polydopamine-Coated PIL Nanovesicles (PDA@PIL)

First, 41.2 mL of the colloidal PIL solution (solid content: 9.7 mg mL^–1^) was diluted into 158.8 mL by water under sonication in 15 min. Then, 0.21g of Tris powder was added into the solution above, and the pH value was adjusted to ∼8.5 with the desired amount of the concentrated HCl solution (37%). After that, 100 mg of dopamine hydrochloride was added to the solution. During the initial dopamine polymerization process, the solution was treated by sonication for 2 h at room temperature. Afterward, it was magnetically stirred at room temperature for 15 h. Finally, the colloidal dispersion of the PDA-coated PIL nanovesicles was purified via dialysis.

### Template Synthesis of Carbon Nanocapsules Functionalized with Iron Nitride Nanoparticles

After dialysis, the volume of the above-mentioned PDA@PIL nanovesicle dispersion was adjusted to 300 mL in a round-bottom flask. After a sonication treatment for 15 min, 0.494 g of potassium ferricyanide (K_3_[Fe(CN)_6_]) was added into the dispersion. Along with sonication treatment for 1 h, the flask was sealed and put into an oil bath at 60 °C for the ion exchange for 24 h. After naturally cooling down to room temperature, it was vacuum filtered off and washed with water three times. The obtained sample was freeze-dried and termed PDA@Fe-PIL. 200 mg of PDA@Fe-PIL powder was placed in an alumina crucible at the center of the tube furnace, and 3 g of melamine powder was placed in another alumina crucible, which was positioned upstream of PDA@Fe-PIL powder. The sample PDA@Fe-PIL was calcinated at 500 °C at a heating rate of 5 °C min^–1^ for 2 h and then naturally cooled down to room temperature under an Ar flow. The obtained sample was termed Fe_*x*_N@C.

### Synthesis of Nanovesicle-Templated Hollow Carbon Nanocapsules and Bulk Carbon Particles

The hollow carbon nanocapsule sample was synthesized by etching off the iron nitride nanoparticles by a HCl solution (4 M) at 60 °C for 15 h. After washing with water and ethanol three times, the etching process was repeated twice to ensure no iron nitride particles were left. Then, the collected hollow carbon nanocapsules were dried at 60 °C under vacuum, termed as “N-Carbon”. The bulk carbon particles (Bulk-Carbon) were obtained by the calcination of PDA@PIL powder under the same condition as that of Fe_*x*_N@C nanocapsules.

### Adsorption Test of Lithium Polysulfides

A Li_2_S_6_ solution was prepared by dissolving stoichiometric sulfur and Li_2_S (5:1, in molar ratio) in a mixed solvent of DOL/DME (1:1, v/v), followed by vigorous stirring at 80 °C for 48 h. Based on the N_2_ adsorption–desorption isotherm analysis, Fe_*x*_N@C and carbon nanoparticle powders with the same specific surface area were added into 6 mL of Li_2_S_6_ solution (1 mM), respectively. After aging for 6 h in a glovebox, the supernatant liquid was centrifuged and sealed in the quartz cuvette for the UV–vis spectroscopy test.

### Kinetics of Li_2_S Precipitation

Initially, the slurry of the host materials (Fe_*x*_N@C or N-Carbon), Ketjen black, and PVDF (8:1:1 weight ratio) in NMP was cast on the carbon paper. After drying at 60 °C under vacuum, the electrode was cut into wafers with a diameter of 14 mm. The areal loading of host materials is around 1 mg cm^–2^. The cell was assembled using 20 μL of Li_2_S_8_ (0.25 M) solution with 1.0 M LiTFSI in the mixture solvent of DOL and DME (1:1, v/v) as catholyte, and 20 μL electrolyte without Li_2_S_8_ was used as anolyte. For the Li_2_S precipitation process, the assembled cells were first discharged galvanostatically at a C-rate of 0.1 C to 2.16 V *vs*. Li/Li^+^ and then discharged potentiostatically at 2.05 V vs Li/Li^+^ for Li_2_S nucleation and growth. The potentiostat discharge was terminated after 65000 s.

### Kinetic Evaluation of Polysulfide Conversion

Two identical electrodes, the same as the one for the Li_2_S precipitation test, were used as the working and the counter electrode, respectively. 40 μL of Li_2_S_6_ (2.5 M, based on sulfur content) solution with 1.0 M LiTFSI in the solvent of DOL and DME (1:1, v/v) was used as electrolyte. The assembled symmetric battery was measured at a scan rate of 10 mV s^–1^ in a potential window from −0.8 to 0.8 V.

### Preparation of the Cathodes

The Fe_*x*_N@C and sulfur powder in a weight ratio of 7:3 were first mixed with the assistance of carbon disulfide. After the solvent was completely evaporated, the mixture was sealed in an autoclave under argon and heated at 155 °C for 12 h. Then, the electrode was prepared by casting the slurry of Fe_*x*_N@C/sulfur, Ketjen black, and PVDF (7:2:1 in weight ratio) on a carbon paper by the doctor blade technique. After drying at 50 °C under vacuum overnight, the electrode was cut into wafers with a diameter of 14 mm. The areal sulfur loading of the electrode is around ∼1.5 mg cm^–2^.

### Electrochemical Measurement

CR2025-type coin cells were assembled with the cathodes, Li foil as the anode, and a piece of Celgard 2700 membrane as the separator in an Ar-filled glovebox (UNIlab plus, M. BRAUN) with H_2_O content <0.5 ppm and O_2_ content <0.5 ppm. One M LiTFSI in DME/DOL (1:1 v/v) with 2 wt % of LiNO_3_ was used as the electrolyte solution. The volume of electrolyte for each cell was 40 μL. Before electrochemical tests, all assembled cells were aged under open circuit potential for 12 h at room temperature to allow the electrolyte to wet the electrode. In this work, the current density of 1 C equals 1675 mA g^–1^. The specific capacity is calculated based on the mass of sulfur content in the electrode. Galvanostatic discharging–charging was conducted on a Neware battery testing system (CT-4008-5 V10 mA) at room temperature. The electrochemical impedance spectroscopy (EIS) was recorded on GAMRY Interface 1000 within a frequency range of 100 kHz to 0.01 Hz. The cyclic voltammetry (CV) curves were measured with a Biologic VMP3 electrochemical workstation with a scanning rate of 0.1 mV s^–1^ in the electrochemical window of 1.7–2.8 V vs Li/Li^+^.

### Density Functional Theory Calculations

Density functional theory (DFT) calculations were performed using the projector augmented wave method that has been implemented in Vienna ab initio Simulation Package. The Perdew–Burke–Ernzerhof exchange–correlation function was adopted, the cutoff energy for this plane-wave basis set was set to be 450 eV, and 5 × 5 × 1 Γ-centered *k*-point grids were used for Brillouin zone integrations.

The (111) crystalline surface of Fe_2_N, Fe_3_N, and Fe_3_N_1.33_ electrodes was modeled by the periodic slab repeated in 2 × 2 surface unit cells. The bottom atom layers in these electrodes were fixed during DFT calculations to simulate bulk structures, while the top atom layers were free to simulate the surface state. A vacuum of 20 Å was contained in each modeling system to reduce interactions between each surface. The isolated Li_2_S_8_, Li_2_S_6_, Li_2_S_4_, and Li_2_S_2_ molecules were modeled in the center of a big enough cubic lattice. All structures were fully relaxed to their optimized geometries with the force convergence set to 0.01 eV/Å. To explore the lowest-energy configurations of adsorbed systems, we carefully manipulated structure parameters of the initial state (the distance, angle, and displacement between molecule and surface) to relax fully and selected the lowest-energy result as the final state for data analysis.

### Material Characterizations

The morphology of the obtained samples was investigated by an LEO 1530 field emission scanning electron microscope (SEM) and a JEOL-2100 transmission electron microscopy (TEM) (JEOL, GmbH, Eching, Germany) operated at 200 kV. X-ray diffraction (XRD) patterns were collected using a Bruker D8 diffractometer with Cu K_α_ radiation. N_2_ adsorption–desorption isotherms were conducted by using Quantachrome Autosorb-1 systems at 77 K. Specific surface areas were calculated using the Brunauer–Emmett–Teller (BET) method based on a multipoint analysis. The chemical states of the elements in the samples were characterized using X-ray photoelectron spectroscopy (XPS) with an ESCA-Lab-220i-XL X-ray photoelectron spectrometer (Thermo Fisher Scientific) with Al K_α_ sources (*h*ν = 1486.6 eV). Thermogravimetric analysis (TGA) was carried out on PerkinElmer (TGA 8000) in the temperature range of 30–900 °C at a heating rate of 10 °C min^–1^ under N_2_ or in synthetic air. UV–vis spectra (300–800 nm) are measured by using Lambda 650 spectrometer supplied by PerkinElmer at 20 °C. Scanning transmission electron microscopy (STEM) characterization employing electron energy-loss spectroscopy (EELS) was performed using a Nion HERMES microscope (Nion Co., Kirkland, WA, USA). Samples for STEM-EELS analysis were prepared by depositing a drop of sample solution on lacey carbon-coated copper TEM grids (200 mesh, Electron Microscopy Sciences, Hatfield, PA) and letting them dry at room temperature. Cryo-TEM was performed with a JEOL JEM-2100 transmission electron microscope (JEOL GmbH, Eching, Germany). Cryo-TEM specimens were vitrified by plunging the samples into liquid ethane using an automated plunge freezer (Vitrobot Mark IV, FEI) set at 4 °C and 95% humidity. Approximately 5 μL of 0.025 wt % solution was deposited on a lacey carbon-coated copper grid (200 meshes, Electron Microscopy Sciences, Hatfield, PA) and equilibrated at the adjusted temperature and humidity for 2 min. After blotting the liquid, the specimens were vitrified, inserted into a precooled cryo-transfer holder (Gatan 914, Gatan, Munich, Germany), and finally transferred into the TEM which was operated at 200 kV. A defocus of the objective lens of about 0.5 μm was used to increase the contrast. Cryo-TEM micrographs were recorded with a bottom-mounted 4 × 4k CMOS camera (TemCam-F416, TVIPS, Gauting, Germany). The total electron dose in each micrograph was kept below 20 e^–^/Å^2^.

## Supporting Information Available

SEM images of the PILs, PDA@Fe-PILs and Fe_*x*_N@C. Photograph of dispersion of PIL vesicles and PDA@PIL vesicles in water. Cryo-TEM images and EDX analysis of the PDA@PIL and PDA@Fe-PIL samples. HR-TEM images of the Fe_*x*_N@C particles. XRD of the PDA@Fe-PIL calcinated at different temperatures. TGA curves of the Fe_*x*_N@C, N-Carbon, Fe_*x*_N@C/S, and N-Carbon/S. TEM images of the PILs-based particles after calcination. Nitrogen adsorption–desorption isotherms of N-Carbon. DFT calculation results of LiPS molecules on different iron nitrides. Charge–discharge curves and EIS spectra of Li–S batteries. TEM image of Fe_*x*_N@C after cycling. Performance comparison of Fe_*x*_N@C/S electrode with other metal nitrides (PDF)
