# Supplementary material for: Poly(ionic liquid) Nanovesicle-Templated Carbon Nanocapsules Functionalized with Uniform Iron Nitride Nanoparticles as Catalytic Sulfur Host for Li–S Batteries
Source: ACS Nano. 2022 Jul 5;16(7):10554–65. doi: 10.1021/acsnano.2c01992 (PMC9331140; doi:10.1021/acsnano.2c01992)
Supplement: Supplementary file 1 — nn2c01992_si_001.pdf [file nn2c01992_si_001.pdf]

Poly(ionic liquid) Nanovesicle-templated Carbon Nanocapsules Functionalized with Uniform Iron Nitride Nanoparticles as Catalytic Sulfur Host for Li-S Batteries

Dongjiu Xie[a,b], Yaolin Xu[a], Yonglei Wang[a], Xuefeng Pan[a,b], Eneli Härk[a], Zdravko Kochovski[a], Alberto Eljarrat[c], Johannes Müller[c], Christoph T. Koch[c], Jiayin Yuan[d], and Yan Lu[a,b]**

AUTHOR ADDRESSES .

[a] Department for Electrochemical Energy Storage, Helmholtz-Zentrum Berlin für Materialien und Energie, Hahn-Meitner Platz 1, Berlin, 14109, Germany

[b] University of Potsdam, Institute of Chemistry, Karl-Liebknecht-Straße 24-25, 14476 Potsdam, Germany

[c] Institut für Physik and IRIS Adlershof, Humboldt-Universität zu Berlin, 12489 Berlin, Germany

[d] Department of Materials and Environmental Chemistry, Stockholm University, Stockholm 10691, Sweden

E-mail: yan.lu@helmholtz-berlin.de, jiayin.yuan@mmk.su.se

Supporting Information

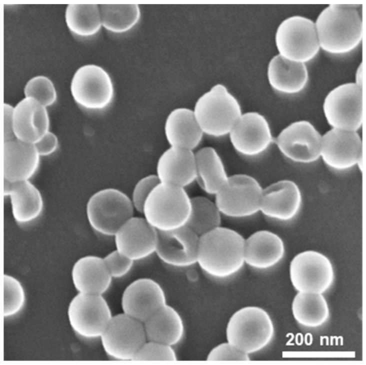

Figure S1. SEM image of the PILs vesicles.

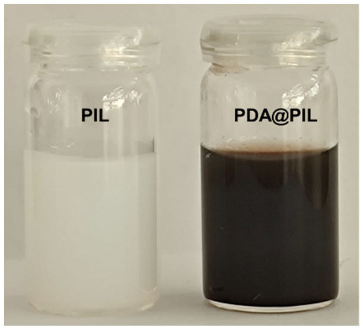

Figure S2. Photograph of dispersion of the PIL vesicles and PDA@PIL vesicles in water.

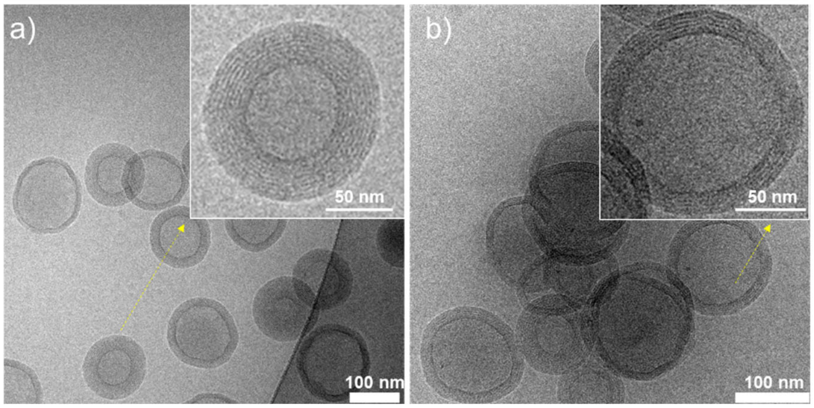

Figure S3. Cryo-TEM images of the PDA coated PIL (a) and PDA@Fe-PIL nanovesicles (b), respectively.

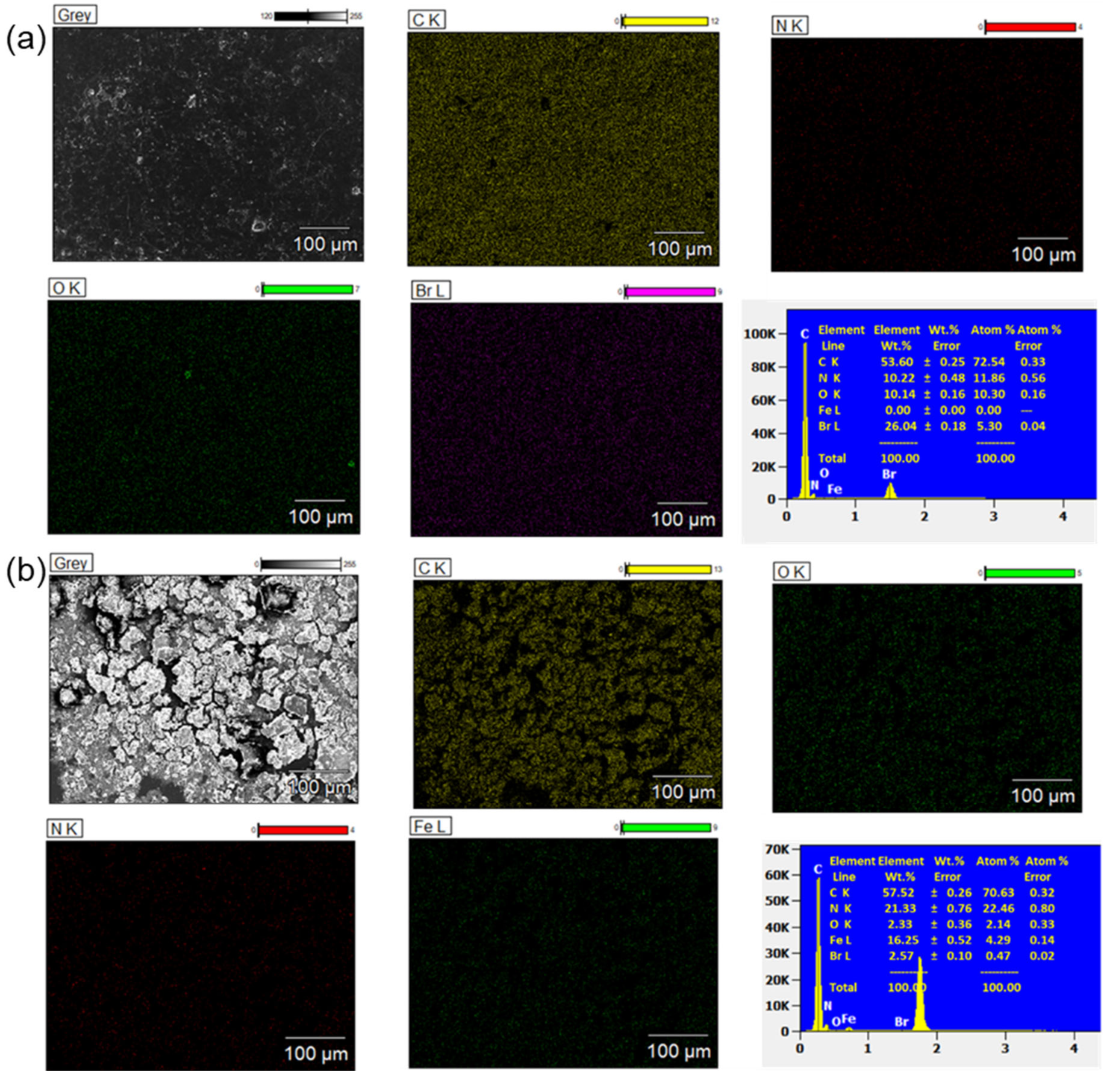

Figure S4. EDX analysis with scanning electron microscope (SEM) images and corresponding element mappings of the PDA coated PIL nanovesicles (a) and the PDA@Fe-PIL nanovesicles (b), respectively.

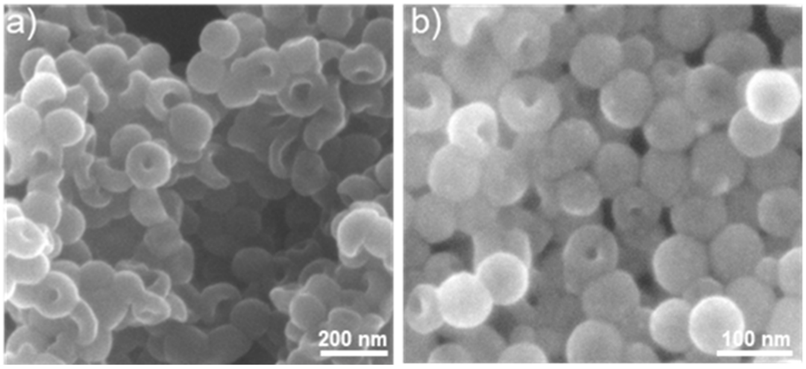

Figure S5. SEM images of the PDA@Fe-PILs vesicles (a) and Fe_xN@C nanocapsules(b).

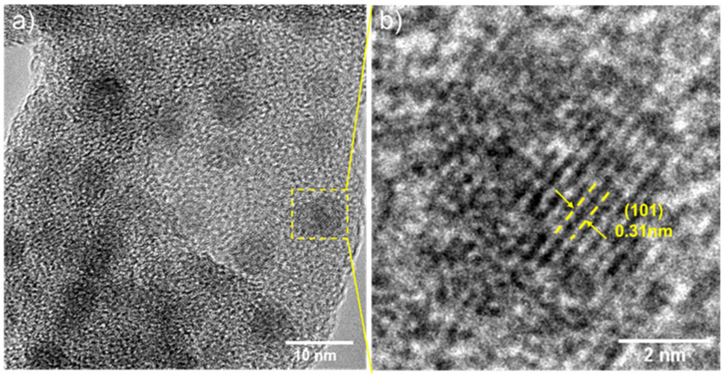

Figure S6. HR-TEM images (a and b) of a randomly selected $Fe_xN@C$ particle.

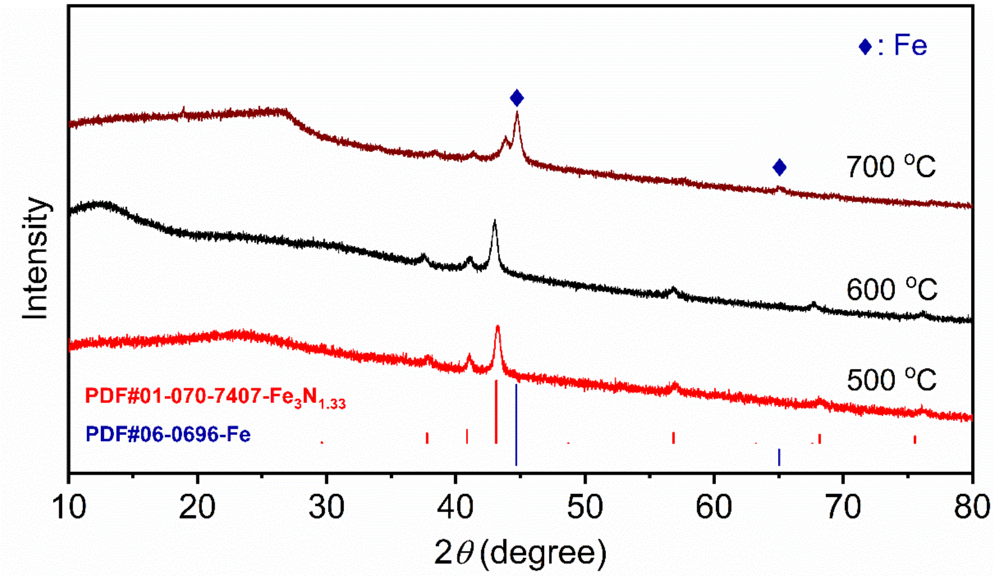

Figure S7. As noted in the figure, XRD patterns of the PDA@Fe-PIL nanocapsules after calcination with melamine at different temperatures.

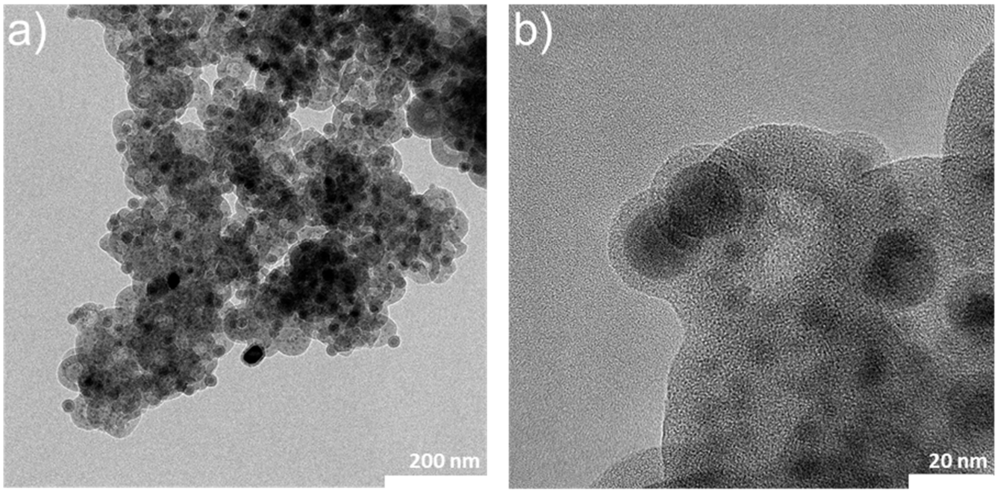

Figure S8. TEM images of the PDA@Fe-PIL nanocapsules after calcination with melamine at 600 ^{o}C for 2 h.

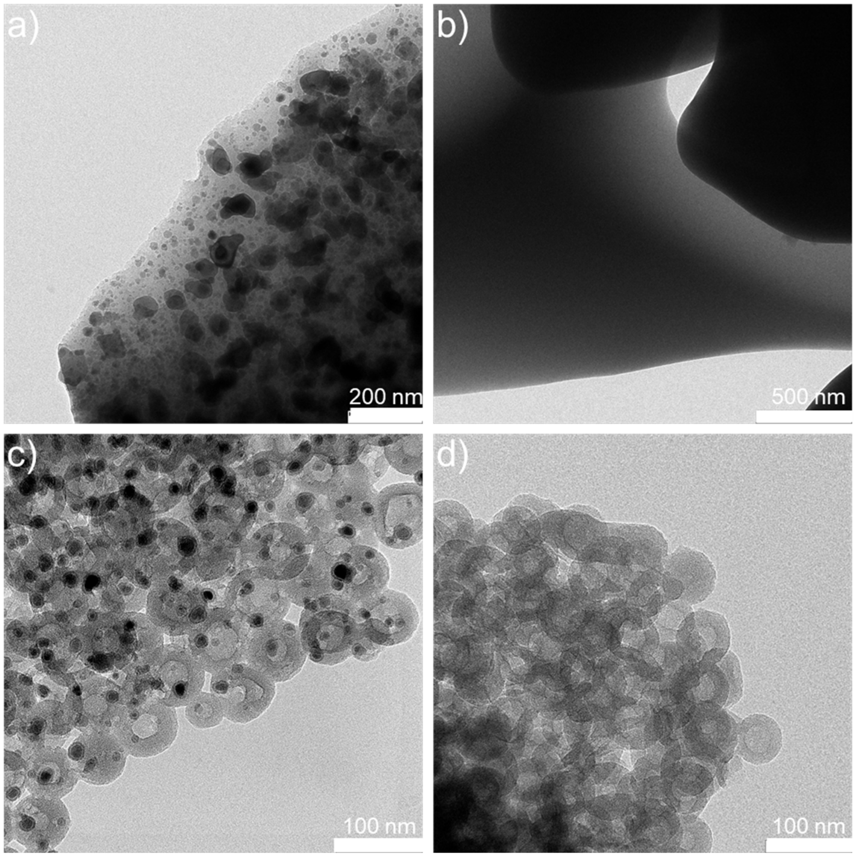

Figure S9. TEM images of the Fe-PIL nanovesicles calcinated at 500 ^{o}C without PDA coating (a), the PDA@PIL calcination without ferricyanide ions, the PDA@Fe-PIL after calcination at 500 ^{o}C without melamine (c), and the hollow N-Carbon nanocapsules (d).

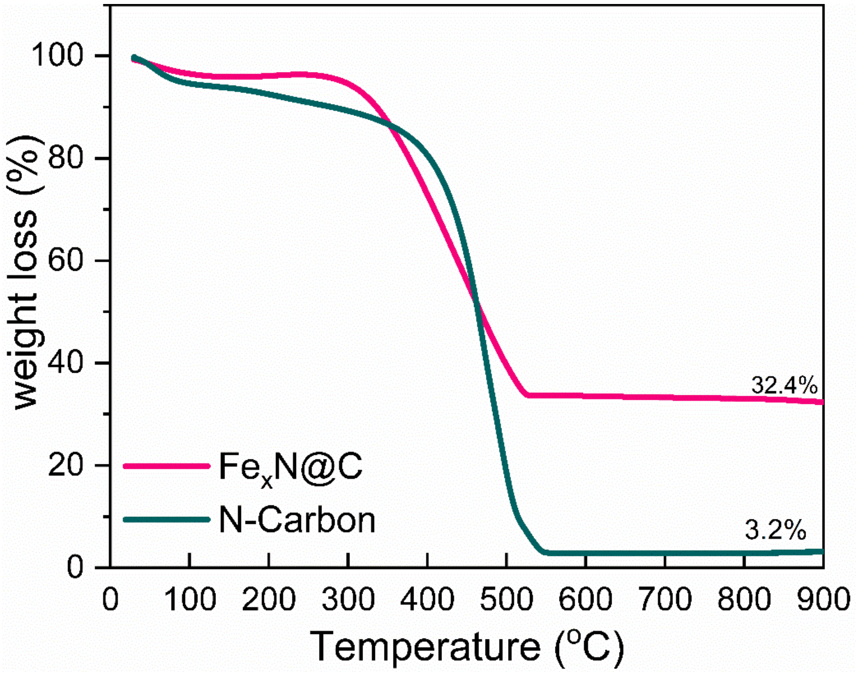

Figure S10. TGA curves of the sample Fe$_x$N@C and N-Carbon nanocapsules in synthetic air.

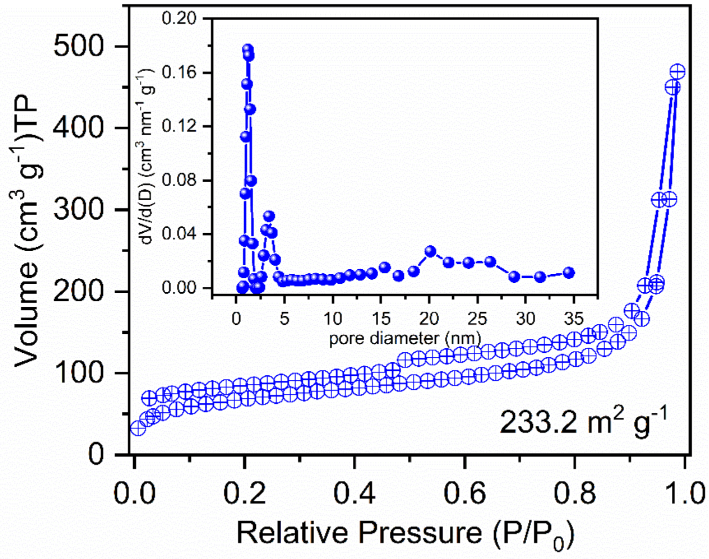

Figure S11. Nitrogen adsorption-desorption isotherms of N-Carbon nanocapsules with the inset corresponding to the pore size distribution plot.

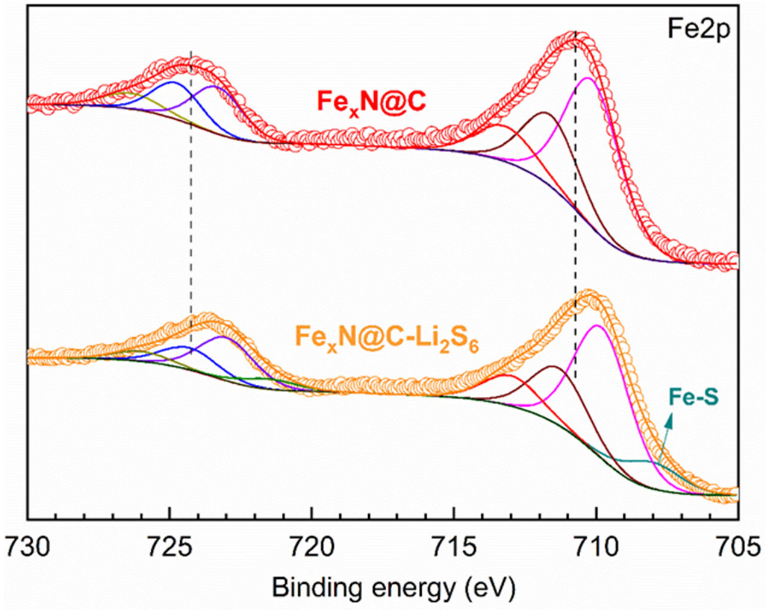

Figure S12. The Fe 2p spectra of the Fe_xN@C particles before and after the adsorption test.

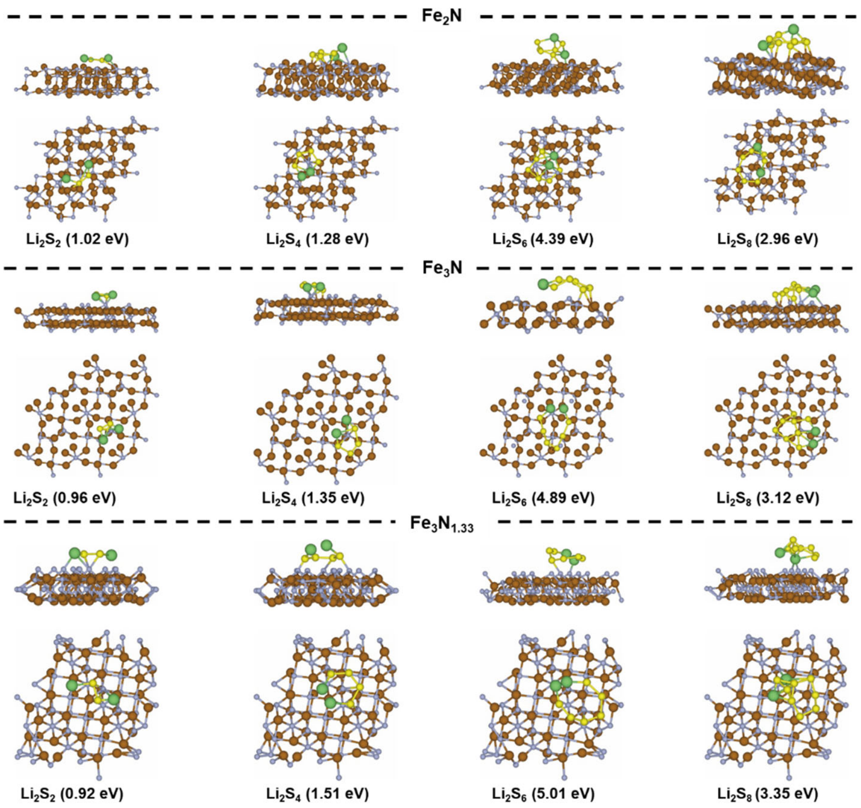

Figure S13. The adsorption configurations and the corresponding adsorption energy of various LiPS molecules (Li_2S_2, Li_2S_4, Li_2S_6, and Li_2S_8) on the different iron nitrides (Fe_2N, Fe_3N, and $Fe_3N_{1.33}$).

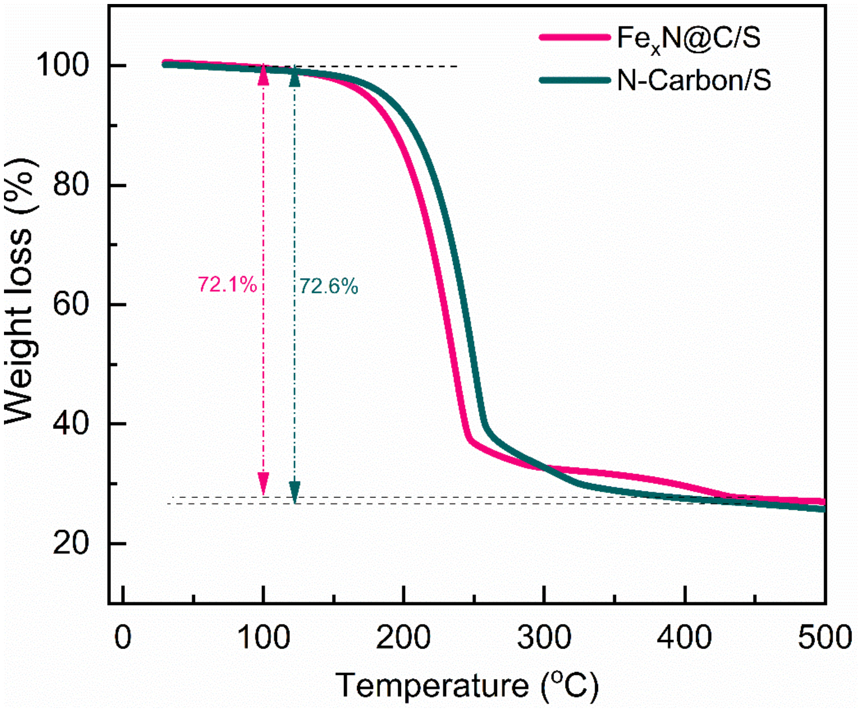

Figure S14. TGA curves of the Fe$_x$N@C/S and N-Carbon/S composites in N_2.

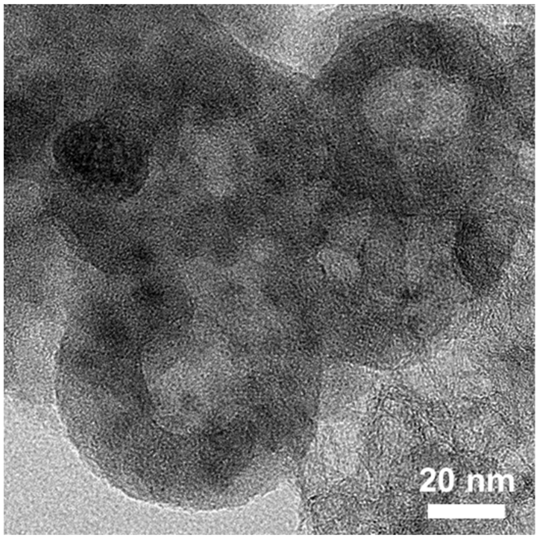

Figure S15. TEM image of the Fe$_x$N@C particles after cycling for 100 cycles at 0.5C.

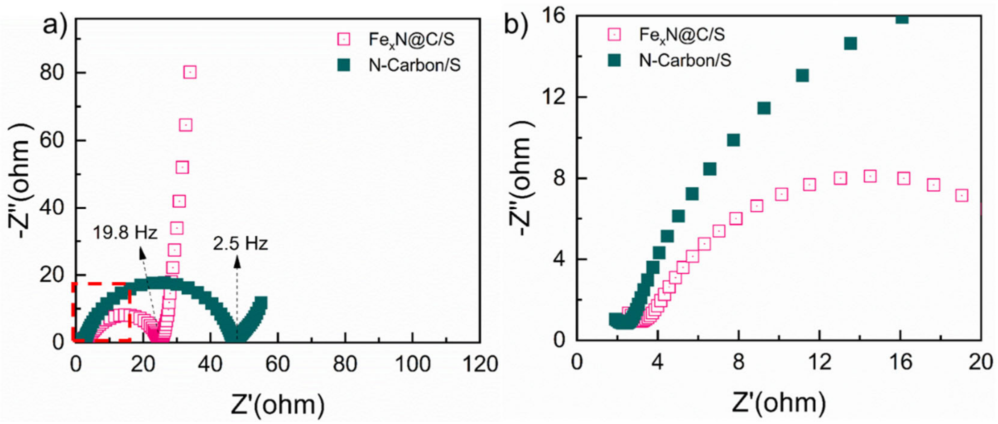

Figure S16. (a) EIS spectra of the Li-S batteries with different cathodes and the corresponding enlarged red rectangle area (b).

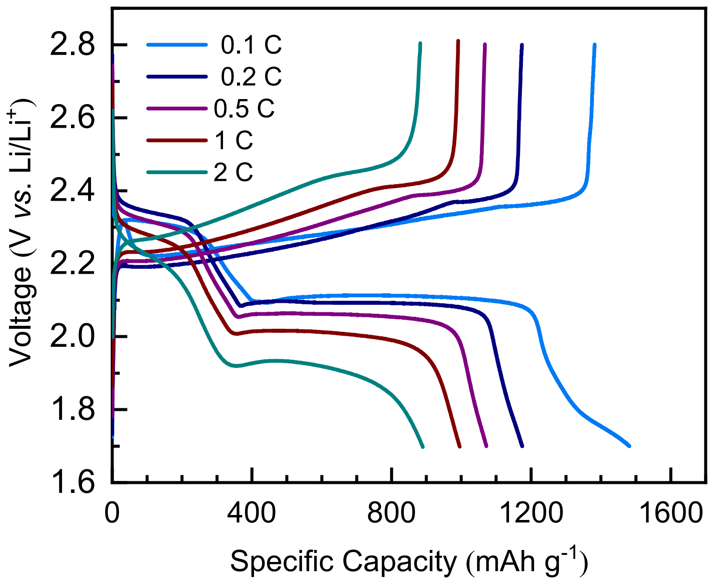

Figure S17. Initial charge-discharge profiles of the Li-S batteries with Fe_xN@C/S electrode at different rates.

Table S1. Performance comparison of the Fe_xN@C/S electrode with other reported metal nitrides as sulfur host for Li-S battery.

Host materials	Areal sulfur loading	Sulfur Content	Current density	Initial Capacity	Ref.
P-Fe_4N@N, P-Graphene	1.1-1.4 mg cm^{-2}	73 wt%	1 C	~850 mAh g^{-1}	S1
VN/rGO xerogels	2.5 mg cm^{-2}	56.6%	1 C<br>2 C	~820 mAh g^{-1}<br>670 mAh g^{-1}	S2
MoO_2–Mo_3N_2	1.2 mg cm^{-2}	70 wt %	1 C	750 mAh g^{-1}	S3
MoN-C-MoN trilayer	1.6 mg cm^{-2}		1 C	765 mAh g^{-1}	S4
$Co_{5.47}N_x$–C	1.8 mg cm^{-2}	86 wt %	0.5C<br>1 C	850 mAh g^{-1}<br>700 mAh g^{-1}	S5
Mesoporous TiN	1.0 mg cm^{-2}.	58.8 wt%	1 C	776 mAh g^{-1}	S6
Co-VN@C	1.3~1.5 mg cm^{-2}	70 wt %	1 C	~800 mAh g^{-1}	S7
NbN@N-Graphene	2.4 mg cm^{-2}	62 wt %	1 C	948 mAh g^{-1}	S8
Ni@Ni_3N@Carbon	1.13 mg cm^{-2}	70 wt %	1C<br>2 C	1047 mAh g^{-1}<br>833 mAh g^{-1}	S9
Co_4N@Carbon Nanocage	1.5 mg cm^{-2}	75.5%	1 C<br>2 C	928 mAh g-1<br>856 mAh g-1	S10
**Fe_xN@C nanocapsules**	1.3~1.5 mg cm^{-2}	72.1 wt %	1 C<br>2 C	**1061 mAh g^{-1}**<br>**890 mAh g^{-1}**	**this work**

References:

S1. M. Zhang, *et al*., Phosphorus-modified Fe_4N@N, P co-doped graphene as an efficient sulfur host for high-performance lithium–sulfur batteries, J. Mater. Chem. A, 2021, 9, 6538-6546

S2. S. Tan, *et al*., Revealing the Origin of Highly Efficient Polysulfide Anchoring and Transformation on Anion-Substituted Vanadium Nitride Host, Adv. Funct. Mater. 2021, 31, 2008034.

S3. R., Li, *et al*., Conductive Holey MoO_2–Mo_3N_2 Heterojunctions as Job-Synergistic Cathode Host with Low Surface Area for High-Loading Li–S Batteries, ACS Nano 2019, 13, 9, 10049-10061

S4. R. Li, *et al*., Sandwich-like Catalyst-Carbon-Catalyst Trilayer Structure as a Compact 2D Host for Highly Stable Lithium-Sulfur Batteries, Angew. Chem. Int. Ed. 2020, 59, 12129.

S5. H. Wu, *et al*., Cobalt nitride nanoparticle coated hollow carbon spheres with nitrogen vacancies as an electrocatalyst for lithium-sulfur batteries, J. Mater. Chem. A, 2020, 8, 14498-14505

S6. Z. Cui, *et al*., Mesoporous Titanium Nitride-Enabled Highly Stable Lithium-Sulfur Batteries, Adv. Mater., 28.32 (2016): 6926-6931.

S7. W. Ren, *et al*., Cobalt-Doped Vanadium Nitride Yolk-Shell Nanospheres @ Carbon with Physical and Chemical Synergistic Effects for Advanced Li–S Batteries, ACS Appl. Mater. Interfaces 2018, 10, 14, 11642-11651

S8. X. Li, *et al*., Conductive Mesoporous Niobium Nitride Microspheres/Nitrogen-Doped Graphene Hybrid with Efficient Polysulfide Anchoring and Catalytic Conversion for High-Performance Lithium-Sulfur Batteries, ACS Appl. Mater. Interfaces 2019, 11, 3, 2961-2969

S9. Y. Li, *et al*., Ni@Ni_3N Embedded on Three-Dimensional Carbon Nanosheets for High-Performance Lithium/Sodium–Sulfur Batteries, ACS Appl. Mater. Interfaces 2021, 13, 41, 48536-48545

S10. Z. Sun, *et al*., Catalytic Polysulfide Conversion and Physiochemical Confinement for Lithium-Sulfur Batteries, Adv. Energy Mater. 2020, 10, 1904010.
